# Bone Benefits of Fish Oil Supplementation Depend on its EPA and DHA Content

**DOI:** 10.3390/nu11112701

**Published:** 2019-11-08

**Authors:** Haissam Abou-Saleh, Allal Ouhtit, Ganesh V. Halade, Md Mizanur Rahman

**Affiliations:** 1 Department of Biological and Environmental Sciences, College of Arts and Sciences, Qatar University, Al Tarfa, Doha PO Box 2713, Qatar; hasaleh@qu.edu.qa (H.A.-S.); aouhtit@qu.edu.qa (A.O.); 2 Division of Cardiovascular Disease, Department of 9 Medicine, University of Alabama at Birmingham, Birmingham, AL 35294, USA; ganeshhalade@uabmc.edu

**Keywords:** omega-3 fatty acids, concentrated fish oil, aging, bone mineral density, inflammation, bone resorption, cytokines

## Abstract

The preventive effect of high-dose (9%) regular-fish oil (FO) against bone loss during aging has been demonstrated, but the effects of a low-dose (1%–4%) of a highly purified concentrated FO (CFO) has not been elucidated. The aim of this study was to determine the dose-dependent effect of a CFO against bone loss in C57BL/6 female mice during aging. Twelve-month old mice were fed with 1% and 4% CFO and 4% safflower oil (SFO) diets, including a group with a 4% regular-FO diet and a group with a lab chow diet for 12 months. Bone mineral density (BMD) was analyzed by dual-energy x-ray absorptiometry (DXA) before and after the dietary intervention. At the end of dietary intervention, bone resorption markers in serum and inflammatory markers in bone marrow and splenocytes and inflammatory signaling pathways in the bone marrow were analyzed. As compared to the 4% SFO control, 4% CFO maintained higher BMD during aging, while 1% CFO offered only a mild benefit. However, the 1% CFO fed group exhibited slightly better BMD than the 4% regular-FO fed group. BMD loss protection by CFO was accompanied by reduced levels of the bone resorption marker, TRAP, and the osteoclast-stimulating-factor, RANKL, without affecting the decoy-receptor of RANKL, osteoprotegerin (OPG). Further, CFO supplementation was associated with an increase in the production of IL-10, IL-12, and IFN-γ and a decrease in the production of TNF-α and IL-6, and the activation of NF-κB, p38 MAPK, and JNK signaling pathways. In conclusion, the supplementation of 4% CFO is very efficient in maintaining BMD during aging, whereas 1% CFO is only mildly beneficial. CFO supplementation starting at middle age may maintain better bone health during aging.

## 1. Introduction

Age-associated bone loss is an eloquent and widespread, health and socioeconomic burden among a fast-growing aging population. Studies reveal that around 50% of women over the age of 50, and 1 out of 4 men, will experience osteoporotic bone fracture [1]. Diminution in bone mineral density (BMD) and increased fracture risks are the major concerns of the aging process and imply new and immediate prevention strategies. The underlying causes for the age-related increase in bone fractures are not clear yet; however, bone resorption seems to play a major role. Despite the unknown etiology of age-related bone loss, one prominent feature is a chronic inflammation reaction driven, in part, by increased pro-inflammatory cytokine expression that contributes to the pathogenesis of the disease. In fact, bone resorption (mediated by osteoclasts) and bone formation (mediated by osteoblasts) are systematically regulated by the interaction of pro-inflammatory and anti-inflammatory cytokines and hormones. Thus, an imbalance in the secretion of these molecules is central to the pathology of osteoporosis.

Despite significant advances in the treatment of osteoporosis, undesirable side effects like cytotoxicity, strange fracture, necrosis of the jaw, etc., and the high cost of treatment may limit their applications. Therefore, it is necessary to develop cost-effective alternatives with fewer undesirable side effects. Dietary therapy and/or lifestyle modifications represent an effective strategy to reduce bone loss. Increasing the intake of omega-3, particularly the long-chain ω-3 polyunsaturated fatty acid (PUFA) may be one such strategy [2]. The most important biologically active ω-3 PUFAs, eicosapentaenoic acid (EPA 20:5) and docosahexaenoic acid (DHA 22:6) are mainly derived from certain cold-water fish or their oils. Because of its health benefits, the use of ω-3 FA supplements is continuously rising worldwide, and 50%–90% EPA/DHA concentrated oils are now commercially available [3]. Omega-3 along with ω-6 PUFAs are essential for optimal health. The Western diet is abundant in ω-6 FA, mainly from linoleic acid (LA 18:2) rich vegetable oils. However, a lower ratio of ω-6/ω-3 fatty acids (FAs) is necessary for the prevention and management of chronic diseases [4]. The arachidonate released from AA serves as the precursor for the synthesis of the biologically active pro-inflammatory eicosanoids, the prostaglandins (PGs), thromboxanes (TXs), and leukotrienes (LTs). On the contrary, dietary ω-3 PUFAs compete with the inflammatory ω-6 PUFAs and displace AA from cell membranes. In addition, EPA and DHA compete with the enzymes that convert AA into pro-inflammatory eicosanoids (PGs, TXs, and LTs) [5,6]. The net effect of increasing dietary consumption of ω-3 PUFAs, relative to ω-6 PUFAs, is to decrease the potential for leukocytes to synthesize potent mediators of inflammation. It has been shown that ω-3 PUFAs can inhibit pro-inflammatory cytokines production and prototypical inflammatory transcription factor NF-κB activation [7]. Most current literature denotes that the ω-6 PUFA AA favors the production of the pro-inflammatory cytokine, while ω-3 FAs can reverse this process in bone cells, thereby promoting bone growth and development [5,8,9,10]. Evidence accumulated over the past 20 years has documented that ω-3 FAs, EPA, and DHA are beneficial for bone health [5,11,12,13,14,15,16,17,18,19,20,21]. We have shown that ω-3 PUFA rich FO prevents bone loss in animal models of post-menopause, arthritis, and aging [11,13,15,22]. FO and low ω-6/ω-3 fatty acids ratio have also been shown to promote bone formation in growing animals [23,24,25,26]. Recently, a strong relationship between higher ω-3 FA intake and improved bone turnover markers and BMD in humans has been reported [27,28,29,30]; however, a few studies have reported no benefit [31]. Very recently, Orchard et al. demonstrated that a higher red blood cell (RBC) ω-3 PUFA was associated with lower fracture risk. On the contrary, a higher RBC ω-6/ω-3 ratio was associated with higher hip fracture in postmenopausal women [32]. 

Clinical studies have shown conflicting results for the efficacy of ω-3 PUFA in osteoporosis, which may have resulted from the varying doses, purity, proportion of EPA and DHA, duration of therapy, and sample size of the population used in the studies. Fish oil enriched with ω-3 FAs are among the most popular dietary supplements used worldwide. EPA and DHA are also added to a vast array of food products. Yet, there have been no reported studies investigating the dose-dependent effect of highly purified fish oil with high content of EPA + DHA on bone loss in mice during aging. Thus, the main objective of this study was to determine the minimal dose of highly purified concentrated fish oil containing 46.5% EPA and 37.5% DHA required for preventing or alleviating osteoporosis in C57BL/6 mice during aging. Moreover, this is the first study to determine the effect of long-term intake of low dose highly purified concentrated fish oil on bone health using C57BL/6 mice starting the supplementation at middle age. Concentrated FO used in the study is an FDA approved prescription drug [33]. Regular FO tested in this study comprised of 18% EPA and 12% DHA. Recent studies revealed that the fish intake-associated protection of bone loss was mainly dependent on the concentration of EPA + DHA consumed [27,34]. Therefore, we hypothesized that a low dose of concentrated FO (CFO) should prevent bone loss during gaining because of its high purity and high content of EPA and DHA. Safflower oil (SFO) was selected as a source of ω-6 fatty acids to underscore the mechanism of increased inflammation and compare against the benefits of low dose concentrated fish oil.

## 2. Materials and Methods

### 2.1. Materials 

CFO (46.5% EPA and 37.5% DHA) was obtained from GlaxoSmithKline, Waltham, MA. The FO-18/12 was generously provided by Ocean Nutrition, Dartmouth, Canada. AIN93M diet ingredients, corn oil (CO), and SFO were purchased from MP Biomedicals (Solon, OH, USA). SFO is a ω-6 fatty acid rich oil and contains 70%–80% ω-6 fatty acids, mainly linoleic acid (LA). 

### 2.2. Animals and Diets

**Figure 1:**
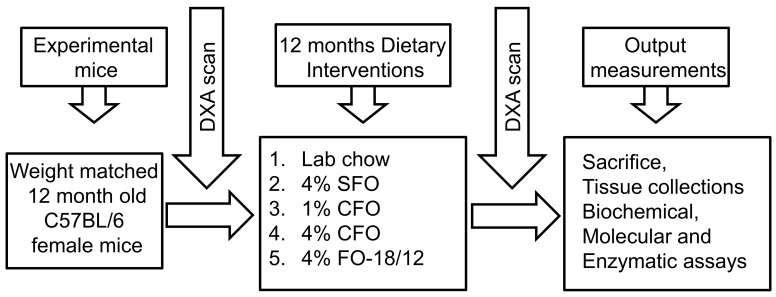
Schematic presentation of the experimental design. Twelve months old C57BL/6 mice were fed AIN93M diets containing 4% safflower oil (SFO), 1%, and 4% concentrated fish oil (CFO) and 4% regular fish oil (FO-18/12). A dual-energy X-ray absorptiometry (DXA) scan was performed at 12-months-old, just before starting the experimental diets to determine the baseline bone mineral density value. Twelve months old C57Bl/6 mice were fed experimental diets for 12 months. Then another DXA scan was performed to determine the endpoint bone mineral density value. Mice were then sacrificed, tissues were collected for further biochemical, molecular, and enzymatic analyses to determine dose-dependent and comparative output measurements of the dietary intervention. SFO, safflower oil containing 70%–80% ω-6 linoleic acid; CFO, concentrated fish oil containing 46.5% EPA and 37.5% DHA; FO-18/12, regular fish oil containing 18% eicosapentaenoic acid (EPA) and 12% docosahexaenoic acid (DHA).

Eleven-months-old female C57BL/6 mice were purchased from Jackson Laboratories (Bar Harbor, ME, USA) and fed a standard lab chow diet (Harlan Teklad LM-485) for 1 month. At 12 months of age, weight-matched mice were divided into 5 groups. Each group consisted of 15 mice (5 mice/cage) and were fed a semi-purified American Institute of Nutrition (AIN)-93 diet containing 4% SFO, 1% CFO, 4% CFO, and 4% FO-18/12, including a group with standard lab chow diet for 12 months. Mice were sacrificed at 24 months of age. In all the diets 1% corn oil (ω-6 fatty acids) was added to prevent essential FA deficiency. As Western diets are abundant with omega-6 fatty acids, omega-6 fatty acids rich safflower oil was used as a control. Lab chow diet was used as an untreated control. The composition of the semi-purified AIN-93 diets is provided in Table 1. One group was fed standard lab chow as a non-FA treatment control. Both the SFO and fish oil diets were supplemented with equal amounts of vitamin E to prevent peroxidation during storage. Fresh diets were prepared weekly and stored in daily served packages. The fresh diet was provided every day in the afternoon, and leftover food was removed daily to prevent rancidity. Animals were fed 5 g diet/mouse. Body weights were monitored routinely on alternate weeks. All the studies were approved by the Institutional Laboratory Animal Care and Use Committee of the University of Texas Health Science Center at San Antonio and all animal procedures were conducted according to the “Guide for the Care and Use of Laboratory Animals” (NIH Publication No. 85–23, revised 1996). The schematic presentation of the experimental design is shown in Figure 1. 

### 2.3. Measurement of BMD

BMD was measured by dual-energy x-ray absorptiometry (DXA) at 12 months of age (baseline value) and at 24 months of age (endpoint value) after 12 months of experimental diets using a Lunar PIXImus mouse bone densitometer (General Electric), and data analysis was carried out manually with PIXImus software as described previously [15]. Percent change in BMD was determined from the endpoint and baseline values for particular bone regions as described earlier [13,15]. 

#### 2.3.1. Serum Bone Turnover Markers Measurement 

Serum receptor activator of NF-κB ligand (RANKL), osteoprotegerin (OPG), and tartrate-resistant acid phosphatase (TRAP)-5b were measured using mouse free soluble RANKL, mouse OPG, and mouse TRAP5b ELISA assay kits from Immunodiagnostic System (IDS) Inc. (Fountain Hills, AZ, USA) according to the manufacturer’s instructions [1].

#### 2.3.2. Isolation of Whole Bone Marrow Cells and Culture

Whole bone marrow (BM) cells were aseptically isolated, as described previously [35]. 

#### 2.3.3. Isolation of Splenocytes and Culture 

Spleens were removed from the mice aseptically and isolated as described previously [36]. Cells (10 × 10^6^ cells/well) were plated in six-well plates, and LPS was added at a concentration of 5.0 μg/mL. After 24 h incubation, the culture medium was collected and analyzed for TNF-α, IL-6, IL-10, IFN-γ, and IL-12 using ELISA kits (eBiosciences, CA), following instructions provided by the manufacturers [36].

### 2.4. NF-κB Activation Assay 

Whole bone marrow cells (5 × 10^6^/well) were plated in 12 well plates and cultured for 3 days in the presence of macrophage colony stimulating factor (MCSF) (30 ng/mL), then stimulated with RANKL (50 ng/mL) or PBS for 30 min. Nuclear proteins were prepared from the cultured cells, as described previously [35]. Protein concentrations of the nuclear extracts were determined using a BCA protein assay kit. NF-κB activation was analyzed using NF-κB transcription factor assay kit (Active Motif, Carlsbad, CA) according to the manufacturer’s instructions [11]. NF-κB -DNA binding was analyzed for NF-κB p65 and NF-κB p50 subunits as described previously [11].

#### 2.4.1. In-Cell Western Analysis for Phospho-JNK and Phospho-p38 

Fast Activated Cell-based ELISA (FACE™) Kits from Active Motif were used to analyze the RANKL stimulated activation of c-Jun N terminal kinase (JNK) and p38 mitogen activated protein kinase (MAPK) in bone marrow cells isolated from the different dietary groups following the manufacturer’s instructions. Briefly, 1 × 10^5^ cells/well were plated in 96 well plates and grown up to 80% confluence in the presence of MCSF (30 ng/mL). Cells were then stimulated with RANKL (50 ng/mL) or PBS for 30 minutes and then fixed with 100 μL of 4% formaldehyde in PBS. Cells were incubated with primary and secondary antibodies and developed. Absorbance at 450 nm was measured on a spectrophotometer (Dynex Technologies, West Sussex, UK) with an optional reference wavelength of 655 nm. The cell density of each well was also determined using crystal violet staining as described in the manufacturer’s instructions. Total and phosphorylated JNK and p38 levels were normalized to cell density.

#### 2.4.2. Statistics 

Data were expressed as means ± SEM. To test the significance either Student’s t-test or a one-way analysis of variance (ANOVA) followed by a Newman-Keuls post hoc test was used. A p-value of <0.05 was considered statistically significant. The analyses were performed using Graphpad prism for Windows (La Jolla, CA, USA).

## 3. Results

### 3.1. Body Weight and Food Consumption 

The average body weights at 24 months of age were not different among the groups (35.77 ± 1.10, 37.28 ± 3.75, 36.69 ± 1.75, 36.51 ± 1.66 and 37.49 ± 1.46 grams for lab chow, 4% SFO, 1% CFO, 4% CFO, and 4% FO-18/12 groups, respectively. The food intake per cage was also monitored. However, there was no difference in food intake among different dietary groups (data not shown). 

### 3.2. Effect of CFO on BMD During Aging 

**Figure 2:**
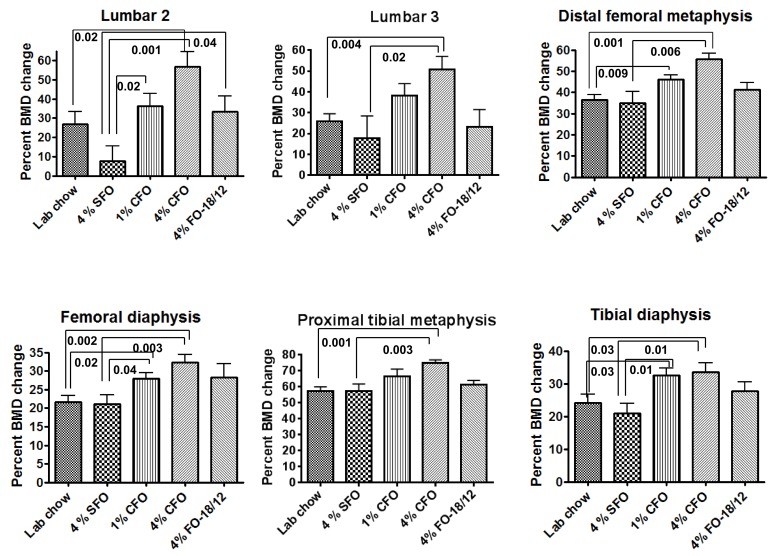
CFO maintains higher levels of bone mineral density during aging. A DXA scan was performed at 12 months of age just before starting the experimental diets to determine the baseline bone mineral density value. Twelve-months-old C57Bl/6 mice were fed experimental diets for 12 months and then another DXA scan was performed to determine the endpoint bone mineral density value. The percent change bone mineral density (BMD) value was analyzed from the endpoint value to the baseline value. Each value represents the mean ± S.E.M. *n* = 8–9 mice per group. *P*-value <0.05 was considered significant by Student’s t-test. The number denotes the p-value. SFO, safflower oil containing 70%–80% ω-6 linoleic acid; CFO, concentrated fish oil containing 46.5% EPA and 37.5% DHA; FO-18/12, regular fish oil containing 18% EPA and 12% DHA.

The 4% SFO fed mice exhibited the lowest BMD in the proximal tibial metaphysis, femoral diaphysis, tibial diaphysis, and the lumbar regions of the spine as compared to that of all the other groups (Figure 2). Interestingly, 4% CFO exhibited the highest BMD in all bone regions as compared to all the other groups. Nevertheless, 1% CFO exhibited significantly higher lumbar, tibial, and femoral BMD than did the 4% SFO group. In addition, 4% regular fish oil exhibited moderately higher levels of BMD in different bone regions as compared to the 4% SFO group. These findings indicated that CFO may maintain higher levels of BMD in C57BL/6 mice during aging. 

### 3.3. Effect of CFO on Serum Bone Turnover Markers During Aging

**Figure 3:**
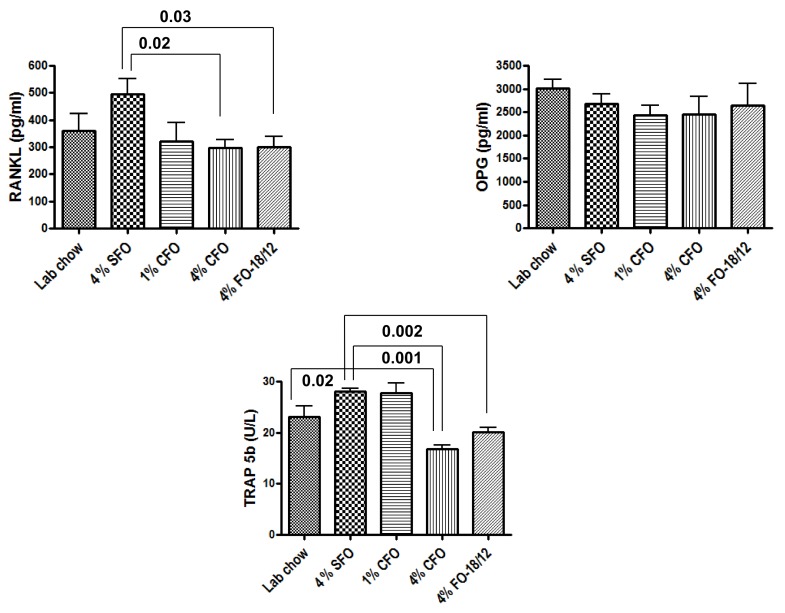
CFO modulates serum bone resorption markers. Twelve-months-old C57BL/6 mice were fed experimental diets for 12 months. Twenty-four-month-old mice were then sacrificed, and serum was collected and analyzed for bone resorption marker TRAP 5b, osteotropic factors RANKL, and its decoy receptor OPG by standard ELISA techniques. Each value represents the mean ± S.E.M. *n* = 4–6 mice per group. *P*-value <0.05 was considered significant by Student’s t-test. SFO, safflower oil containing 70%–80% ω-6 linoleic acid; CFO, concentrated fish oil containing 46.5% EPA and 37.5% DHA; FO-18/12, regular fish oil containing 18% EPA and 12% DHA.

The RANKL, OPG, and TRAP-5b was measured in the serum samples collected at the time of sacrifice. RANKL is one of the most critical osteoclastogenic factors [37], and serum TRAP5b level indicates the current status of osteoclast function. OPG is a decoy receptor for RANKL. Interestingly, it was found that both serum RANKL and TRAP5b levels were significantly lower in 4% CFO, and 4% regular FO fed mice as compared to the 4% SFO fed group. However, serum OPG levels were not different among the groups (Figure 3). The results indicated that 4% CFO may prevent aging associated bone loss by modulating osteoclastogenesis and bone resorption. 

**Figure 4:**
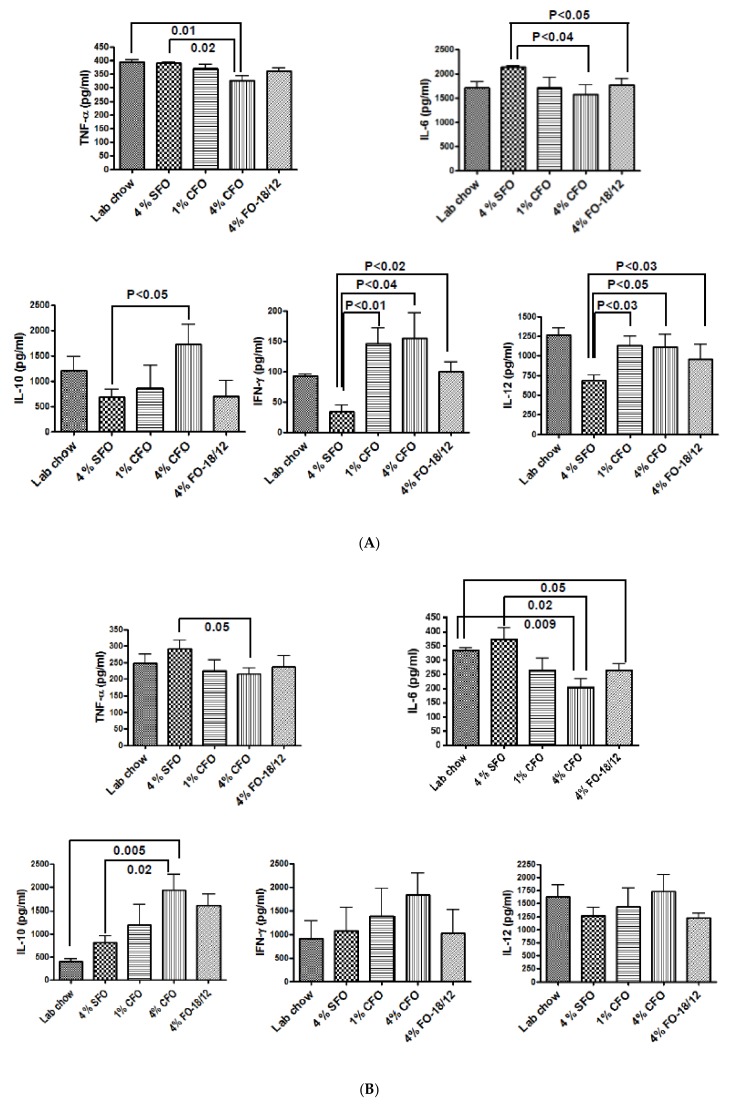
CFO modulates LPS stimulated cytokines production. Twelve-months-old C57BL/6 mice were fed experimental diets for 12 months. Twenty-four-months-old mice were then sacrificed, and bone marrow cells (**A**) and splenocytes (**B**) were isolated and cultured and then stimulated with 5 µg/mL LPS. After 24 hrs, culture media were collected and analyzed for TNF-a, IL-6, IL-10, IL-12 and IFN-g by standard ELISA techniques. Each value represents the mean ± S.E.M. of two independent triplicate cultures. *P*-value <0.05 was considered significant by Student’s t-test. SFO, safflower oil containing 70%–80% ω-6 linoleic acid; CFO, concentrated fish oil containing 46.5% EPA and 37.5% DHA; FO-18/12, regular fish oil containing 18% EPA and 12% DHA.

The effect of CFO on LPS stimulated cytokine production by bone marrow cells and splenocytes. Whether the BMD loss protection observed in 4% CFO had an impact on inflammation-related cytokines expression was examined. Pro-inflammatory cytokines such as IL-6 and TNF-α are key regulators of osteoclastogenic activity and have been shown to increase bone resorption [38]. Interestingly, a significant increases in TNF-α and IL-6 production by LPS stimulated BM and splenocytes from the 4% SFO fed aged mice was found. However, LPS-stimulated BM and splenocytes from the 4% CFO group exhibited significantly lower levels of these pro-inflammatory cytokines as compared to the 4% SFO group. However, 1% CFO and regular FO exhibited moderately lower levels of pro-inflammatory cytokine production by LPS stimulated BM and splenocytes as compared to the 4% SFO (Figure 4A,B). IL-10 is reported to inhibit bone resorption in inflammatory disorders [39]. LPS stimulated anti-inflammatory cytokine IL-10 production by both BM and splenocytes was significantly higher in the 4% CFO group as compared to the 4% SFO group. A moderate increase in the production of IL-10 was observed both in the 1% CFO and the 4% regular FO groups as compared to the 4% SFO group. IFN-γ and IL-12 are known to be strong suppressors of osteoclastogenesis [40,41]. Significantly increased production of both IFN-γ and IL-12 by LPS stimulated BM cells from the 4% CFO group as compared to the 4% SFO group was found. A moderate increase in the production of IFN-γ and IL-12 by LPS-stimulated BM cells was observed both in the 1% CFO and the 4% regular FO groups as compared to the 4% SFO group. However, there was no significant difference in IFN-γ and IL-12 production by LPS stimulated splenocytes among the groups. These data indicate that CFO probably maintains better BMD by suppressing bone resorption due to reduced production of pro-inflammatory pro-osteoclastogenic cytokines and increased production of anti-inflammatory, anti-osteoclastogenic cytokines. 

### 3.4. Effect of CFO on RANKL-Stimulated Activation of NF-κB, JNK, and p38 MAPK 

**Figure 5:**
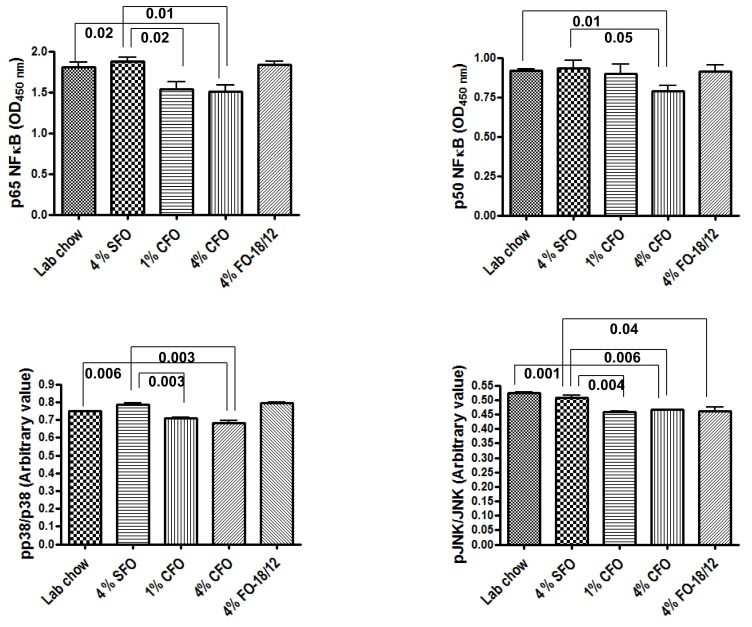
CFO suppresses RANKL-stimulated activation of NF-kB, p38 MAPK, and JNK signaling. Twelve-months-old C57Bl/6 mice were fed experimental diets for 12 months. Twenty-four-months-old mice were then sacrificed, and bone marrow cells were isolated and cultured in 24 well plates in the presence of 30 ng/mL MCSF for three days and then stimulated with 50 ng/mL RANKL for 30 minutes, and then nuclear proteins were prepared. 10 µg of nuclear proteins were analyzed for p65 NF-kB and p50 NF-kB-DNA binding activity using TransAM Transcription Factor Assay kit. Activation of p38 and JNK were analyzed using in cell Western phosphor ELISA system. Bone marrow cells are isolated and cultured in 96 well plates in the presence of MCSF for three days, followed by stimulation with RANKL for 30 minutes and analyzed for the total and phosphor protein levels according to the manufacturer’s instructions. Phospho and total proteins were normalized with cell density according to the manufacturer’s instructions. Then phosphoprotein levels were normalized with total protein levels. Each value represents the mean ± S.E.M. of two independent triplicate cultures. *P*-value <0.05 was considered significant by Student’s t-test. SFO, safflower oil containing 70%–80% ω-6 linoleic acid; CFO, concentrated fish oil containing 46.5% EPA and 37.5% DHA; FO-18/12, regular fish oil containing 18% EPA and 12% DHA.

The effects of CFO on the activation of inflammatory signaling pathways like NF-κB, JNK, and p38 MAPK was examined. Activation of these signaling pathways was associated with enhanced osteoclastogenesis and related bone resorption [35,42]. Activation of these inflammatory signaling pathways was also associated with enhanced production of pro-inflammatory cytokines [43]. On the other hand, inflammatory cytokines can also activate these signaling pathways [44]. We examined if the RANKL stimulated activation of NF-κB, JNK, and p38 MAPK in BM cells was modulated by CFO. The values of the PBS treated group was used to ensure RANKL stimulated activation of NF-κB, JNK and p38 MAPK in BM cells (data not shown). BM cells from both 1% and 4% CFO stimulated with RANKL exhibited significantly lower activation of NF-κB, JNK and p38 MAPK as compared to that of 4% SFO (Figure 5). However, regular FO group exhibited significant reduction of RANKL stimulated activation of JNK as compared to 4% SFO. These data indicate that CFO inhibits the activation of pro-inflammatory, pro-osteoclastogenic signaling pathways leading to reduction in inflammation in bone microenvironment, thereby protecting aging-associated bone destruction by excessive bone resorption.

## 4. Discussion

We have previously reported that a diet containing 9% regular FO (EPA/DHA, 18%/12%) could prevent bone loss during aging [13]. Nevertheless, there are significant concerns that diets supplemented with such a high volume of FO may be an impractical dose for human consumption. Recently, highly purified concentrated fish oil, CFO, which contains 46.5% EPA and 37.5% DHA became available and has been approved by the FDA. Therefore, we attempted to determine if human achievable low dose concentrated FO can maintain higher BMD during aging. In fact, 4% CFO supplementation in mice per day could supply a higher amount of EPA and DHA than that of 9% regular FO. In this study, in addition to the 4% CFO, 1% CFO was also used to investigate a possible dose response. A previous study demonstrated that high intakes (≥3 servings/week) of omega-3 rich fish were associated with maintenance of femoral neck BMD in humans [27]. This bone protective effect was associated with a higher intake of EPA and DHA (ω-3 FA). They also showed that linoleic acid (LA) (ω-6 FA) intake was associated with femoral neck-BMD loss in women. Omega-6 FAs are known to induce various inflammatory pathological conditions [45,46]. In animal models, ω-3 FA deficiency caused severe osteoporosis [47], whereas higher ω-3 FA intake was associated with lower bone resorption [48]. These findings suggest that improvement of bone health might be mainly associated with the quantities of EPA and DHA that is supplemented. In this study, 12 month old mice, which are equivalent to 35 years old of human (middle age) were fed with experimental diets up to 24 months of age, which is equivalent to a 70 years old of human (elderly) to determine if concentrated FO can maintain BMD during aging. Our study demonstrates that 4% CFO maintains higher levels of BMD while 1% CFO offers only mild benefit during aging. However, slightly better BMD levels were observed in the 1% CFO group than in the 4% regular-FO group, despite having a lower content of EPA and DHA. This effect might be because of the purity of the product. The higher levels of BMD in the femur, tibia, and lumbar regions of the 4% CFO fed mice was accompanied by a lower incidence of bone resorption markers. At the molecular level, these changes were accompanied by lower activation of NF-κB, p38 MAPK and JNK, and a decrease in pro-osteoclastogenic TNF-α, IL-6, and an increase of the anti-osteoclastogenic cytokines IL-10, IL-12 and IFN-γ production. These data support our previous findings that ω-3 FA, either endogenously converted or supplemented, can decrease osteoclastogenesis [11,13]. Thus, CFO intake is probably preventing bone loss during aging by attenuating osteoclastogenesis. For the first time, the present study revealed the dose-dependent effect of long-term supplementation of low dose concentrated fish oil on bone health in mice during aging. 

Biomarkers of bone turnover can be used as a non-invasive assessment of skeletal pathology. Long term treatment with 4% CFO reduced the levels of bone resorption marker TRAP5b. These data suggest that CFO may protect bone loss during aging by suppressing bone resorption, either by directly inhibiting osteoclast formation or by interfering with the function of mature osteoclasts. Our results also showed that CFO inhibited osteoclastogenic factor RANKL, which is essential for the differentiation of pre-osteoclasts to mature osteoclasts, and activation of osteoclast function. However, CFO did not affect the decoy receptor of RANKL, OPG production. Therefore, CFO may prevent both differentiation and activation of osteoclast function, thereby decreasing bone resorption leading to reduction of bone loss in aging mice. 

The levels of pro-inflammatory cytokines IL-1β, IL-6, and TNF-α, are increased with aging because they play a major role in bone remodeling [13]. Moreover, findings from both in vitro and in vivo studies support the role of these factors in the pathogenesis of osteoporosis [49,50]. These mediators activate differentiation of bone-resorbing cells, leading to accelerated bone resorption [38,51]. Increased levels of these cytokines increase the risk of hip fractures in older women [49,50]. Furthermore, inflammatory cytokines induce the expression of COX-2 in osteoblastic and stromal cells, resulting in an increased production of an osteoclastogenic factor, PGE2 [52]. Our present study showed lower levels of TNF-α and IL-6 in CFO-fed mice, indicating the maintenance of higher BMD in these mice. In fact, we have previously demonstrated that FO and ω-3 FA decreased the expression and activity of these cytokines, both in vivo and in vitro [11,13,53,54,55]. TNF-α and IL-6 promote bone resorption by increasing osteoclast differentiation [56]. Thus, a decreased activity of IL-6 and TNF-α may also explain the lower level of the bone resorption marker, TRAP, in the CFO-fed mice observed in our study. Our findings also showed a significant increase in LPS-stimulated production of IL-10 in BM cells. IL-10 plays a critical role in in vivo regulation of pro-inflammatory cytokine levels and has been reported to suppress osteoclastogenesis [57,58]. The heterodimers p50 and p65 NF-κB are intimately involved in the activation of inflammatory genes by IL-1 or TNF-α in human monocytes. These effects are blocked by the anti-inflammatory cytokine IL-10 [59]. Thereby, up-regulation of anti-inflammatory cytokines may also be one of the mechanisms by which CFO suppresses osteoclastic bone resorption. IFN-γ derived from TH1 cells is also known to suppress osteoclast differentiation [40]. The suppressive function of IFN-γ on osteoclast differentiation is based on the interference of RANKL signaling, where IFN-γ constitutes a negative feedback loop for RANKL-mediated osteoclast activation [40]. In this study, we showed an up-regulation of LPS-stimulated IFN-γ production in BM cells from the CFO treated mice. IL-12, the major differentiation signal for TH1 cells, is also known to inhibit osteoclastogenesis [41]. Interestingly, our findings revealed an increase in LPS-stimulated IL-12 production in BM cells isolated from the CFO treated-mice, as compared to that of the 4% SFO fed-mice. These data put together indicate that CFO may protect against bone loss during aging by suppressing osteoclastic bone resorption mediated by inflammatory cytokines.

Activation of the inflammatory signaling pathways is a key process in accelerated osteoporotic bone resorption during aging, and the role of NF-κB in the pathogenesis of osteoporosis is well documented. NF-κB null mice develop osteopetrosis and contain very few osteoclasts compared to normal controls, indicating the essential role of NF-κB signaling pathway in osteoclast generation and activation [42,60]. However, constitutive expression of intestinal NF-κB does not trigger destructive inflammation unless it is accompanied by MAPK activation [61]. The p38 MAPK pathway also regulates bone resorption, induced by estrogen deficiency, and selective inhibitors of this pathway can prevent bone loss in postmenopausal osteoporosis [62]. Activation of p38 MAPK and JNK is also required for osteoclastogenesis [35]. In fact, a recent study showed that suppression of JNK1 enhanced BMP2-stimulated osteoblastogenesis [63]. It is now clear that ω–3 FAs might exert their effects on inflammatory gene expression via direct actions on the intracellular signaling pathways. We have in fact previously showed that ω–3 FA down-regulated the activity of NF-κB, a key regulator of inflammatory pathways [11,22]. Omega-3 FA can also decrease endotoxin-induced activation of NF-κB and MAPK in human monocytes [64,65]. Moreover, fish oil can inhibit LPS induced NF-κB activation in macrophage [66,67,68], suggesting a direct effect of ω–3 FA on inflammatory gene expression through inhibition of NF-κB. In this study, reduced NF-κB and MAPK activation in BM cells isolated from CFO fed mice compared to those of the 4% SFO control mice, was observed. These data support the notion that the NF-κB and MAPK pathways are essential in age-associated osteoporosis, and are, therefore, potential targets for intervention in osteolytic processes. More interestingly, CFO may prevent osteoporosis during aging by attenuating bone resorption via suppression of the NF-κB and MAPK signaling pathways.

## 5. Conclusions 

**Figure 6:**
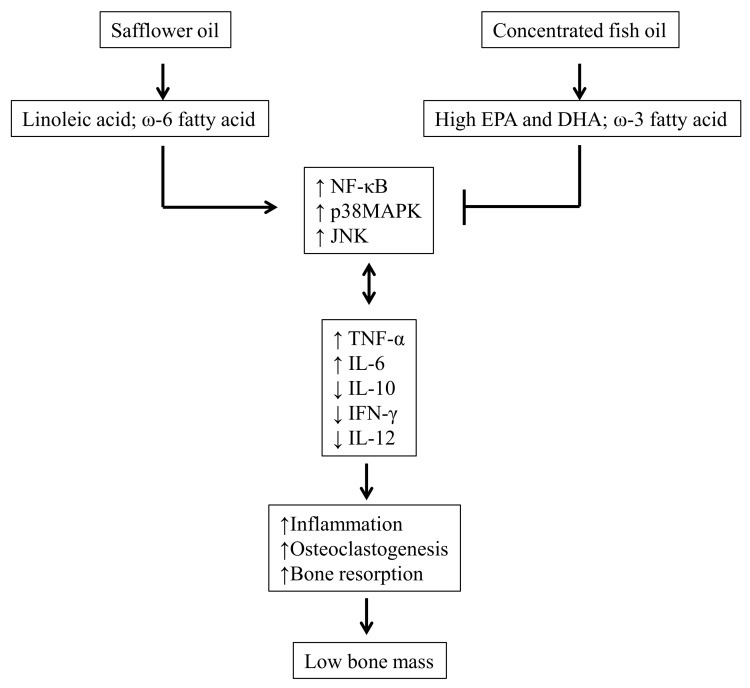
Possible mechanisms of bone loss protection by EPA and DHA enriched concentrated fish oil.

Our current findings put together support the hypothesis that a low dose of CFO with a high content of EPA and DHA might protect against bone loss in C57BL/6 mice during aging. Possible mechanisms of bone loss protection by EPA and DHA enriched fish oil is shown in Figure 6. CFO protects bone loss during aging, primarily by inhibiting inflammation-associated with bone resorption. The mice fed with 4% CFO maintained a better BMD than 1% CFO, supporting the notion that the FO-mediated beneficial effect on bone health is associated with the concentration of EPA + DHA supplemented. Thus, CFO may be beneficial in preventing bone loss during aging. Further studies are necessary to determine if CFO has any therapeutic value in treating osteoporosis. 

## Figures and Tables

**Table 1:** Composition of AIN-93 semi-purified diets containing safflower oil (SFO), concentrated fish oil (CFO) and regular fish oil (FO-18/12).

^a^Diet ingrediants	4% Placebo	1% Lovaza	4% Lovaza	4% FO-18/12
Casein	14.00	14.00	14.00	14.00
Corn starch	47.43	50.43	47.43	42.73
Dextronized corn starch	14.50	14.50	14.50	14.50
Sucrose	9.00	9.00	9.00	9.00
Cellulose	5.00	5.00	5.00	5.00
AIN-93 mineral mix	3.50	3.50	3.50	3.50
AIN-93 vitamin mix	1.00	1.00	1.00	1.00
L-cysteine	0.18	0.18	0.18	0.18
Choline bitartrate	0.25	0.25	0.25	0.25
TBHQ	0.10	0.10	0.10	0.10
Vitamin E	0.04	0.04	0.04	0.04
Corn oil	1.00	4.00	1.00	1.00
SFO (Safflower oil)	4.00	0.00	0.00	0.00
^b^Lovaza	0.00	1.00	4.00	0.00
^c^Fish oil-18/12	0.00	0.00	0.00	4.00

^a^ All diet ingredients (expressed as percent total diet) were purchased from MP Biomedicals (Irvine, CA, USA). ^b^ CFO (concentrated fish oil) was obtained from Glaxo Smithkline (Waltham, MA, USA). ^c^ FO-18/12 (18% Eicosapentaenoic acid and 12% Docosahexaenoic acid) was supplied by Ocean Nutrion, Dartmouth, Canada).
